# Real-world performance of postpericardiotomy syndrome diagnostic criteria—An analysis of the prospective Cardiovascular Research Consortium—A Prospective Project to Identify Biomarkers of Morbidity and Mortality in Cardiovascular Interventional Patients (CAREBANK) database

**DOI:** 10.1016/j.xjon.2026.101844

**Published:** 2026-04-28

**Authors:** Nea Vasarainen, Joonas Lehto, Jarmo Gunn, Maija Hollmen, Tuomas O. Kiviniemi

**Affiliations:** a Heart Center, Turku University Hospital and University of Turku, Turku, Finland; b MediCity Research Laboratory, Department of Microbiology and Immunology, InFLAMES Flagship, University of Turku, Turku, Finland

**Keywords:** postpericardiotomy syndrome, thoracic surgery, diagnosis, mortality, pericardium

## Abstract

**Objectives:**

Postpericardiotomy syndrome (PPS) is a well-recognized postoperative complication in cardiac surgery. Current diagnostic criteria, however, seem to lack specificity and fail to account for common confounding factors encountered in clinical settings.

**Methods:**

We examined the performance of current PPS diagnostic criteria in a prospective cohort of 1001 patients from the CAREBANK (Cardiovascular Research Consortium—A Prospective Project to Identify Biomarkers of Morbidity and Mortality in Cardiovascular Interventional Patients) database who underwent cardiac surgery between 2016 and 2021 at a tertiary center. Considering clinical relevance together with the evaluation of coexisting confounding conditions, the patients fulfilling the criteria were divided into 4 categories: “definite PPS,” “likely PPS,” “unlikely PPS,” and “no PPS.”

**Results:**

The incidence of “definite PPS” and “likely PPS” was 4.0% each, whereas “unlikely PPS” and “no PPS” accounted for 8.7% and 83.3%, respectively. The median time to diagnosis was 42 days (interquartile range, 19-63) days for “definite PPS” and 24 days (9-49) days for “likely PPS.” Patients categorized as “likely PPS” or “unlikely PPS” exhibited greater European System for Cardiac Operative Risk Evaluation II values, increased incidence of new-onset atrial fibrillation, delayed postoperative ventilation, and postoperative stroke, and elevated first-year mortality.

**Conclusions:**

Accurate diagnosis of PPS is often challenged by overlapping clinical conditions, especially in patients with multiple comorbidities. The majority of patients fulfilling current PPS diagnostic criteria have confounding explanations rather than genuine inflammatory disease. The presented 4-tier approach enhances the accuracy of distinguishing true inflammatory PPS and conditions that mimic its clinical presentation and should therefore be considered in future studies.

**Figure:**
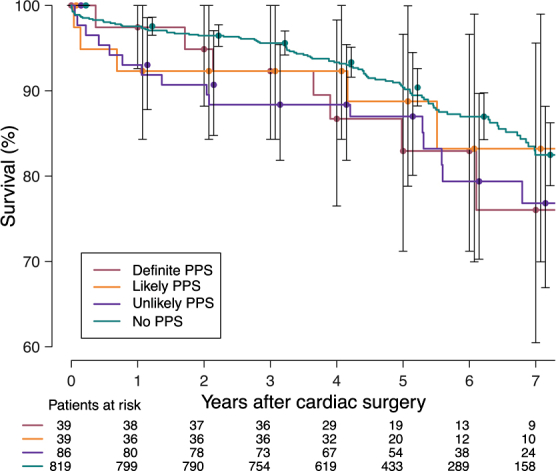
Survival in different postpericardiotomy syndrome (PPS) categories.

**Central Message**

In a prospective real-world setting, the majority of patients fulfilling current PPS diagnostic criteria have confounding explanations rather than genuine inflammatory disease.

**Perspective**

There is a clinical need for more refined diagnostic criteria for PPS, consistent with real-world practice. Only half of patients meeting the ESC criteria were classified as either ‘Definite PPS’ or ‘Likely PPS,’ underscoring the limitations of current diagnostic standards in routine clinical practice. The presented four-tier approach enables the more specific identification of true inflammatory PPS.

Postpericardiotomy syndrome (PPS) results in hospital readmissions, longer hospital stays, and invasive interventions such as the evacuation of pericardial or pleural effusion after cardiac surgery. The prognosis of the syndrome has traditionally been regarded as benign.^1^, ^2^, ^3^ However, recent epidemiologic data have demonstrated an association between PPS and increased mortality within the first postoperative year, thereby challenging the previously held notion of its benign nature.^4^^,^^5^ In studies conducted over the past decade, the reported incidence of PPS in adult patients ranged from 9% to 21%.^5^, ^6^, ^7^, ^8^, ^9^, ^10^, ^11^ Recent prospective studies have reported the greatest incidence rates, ranging from 21 to 29%.^7^^,^^12^ However, PPS appears significantly less prevalent in the real-world clinical practice, with an incidence of approximately 9 to 11%.^5^^,^^10^ Consequently, most of the PPS diagnoses reported in previous prospective studies are clinically irrelevant.^10^ Although the European Society of Cardiology (ESC) has released standardized diagnostic criteria for the syndrome,^13^ the recommended criteria have received criticism because of their lack of specificity.

This study evaluated the real-world performance of current PPS diagnostic criteria and their reliability in the presence of confounding conditions. In addition, we examined the predictors across different PPS groups, as well as associated complications, treatment, and prognosis.

## Methods

### Data Collection

The present analysis includes a prospective cohort of 1001 consecutive patients who were undergoing adult cardiac surgery from February 2016 to December 2021 at the Heart Center of the Turku University Hospital, Turku, Finland. These patients are participants from a database included in the CAREFIB Consortium: CAREBANK (Cardiovascular Research Consortium—A Prospective Project to Identify Biomarkers of Morbidity and Mortality in Cardiovascular Interventional Patients; ClinicalTrials.gov Identifier: NCT03444259). The CAREFIB Consortium is part of a broader ongoing protocol in Finland aimed at evaluating thromboembolic and bleeding complications related to AF management in patients undergoing cardiac procedures.

The CAREBANK is an ongoing Finnish prospective cohort study assessing the associations between the features of cardiac tissue samples and clinical phenotypes in patients undergoing cardiac procedures. As part of the study protocol, all major adverse events including PPS, atrial fibrillation (AF), cerebrovascular events, and myocardial infarctions are collected prospectively both by individual contacts by phone calls using a structured questionnaire and from hospital records at prespecified time points. The structured data-collection protocol includes pre- and perioperative data, discharge data, and long-term follow-up events. Mortality data were obtained from patient records as well as from Statistics Finland. This governmental office monitors the time and causes of death in Finland. Personal and immutable social security numbers are used to identify the patients. Therefore, each case is carefully monitored even if the person moved. The end points of this prespecified study included the occurrence of PPS and death.

**Figure 1:**
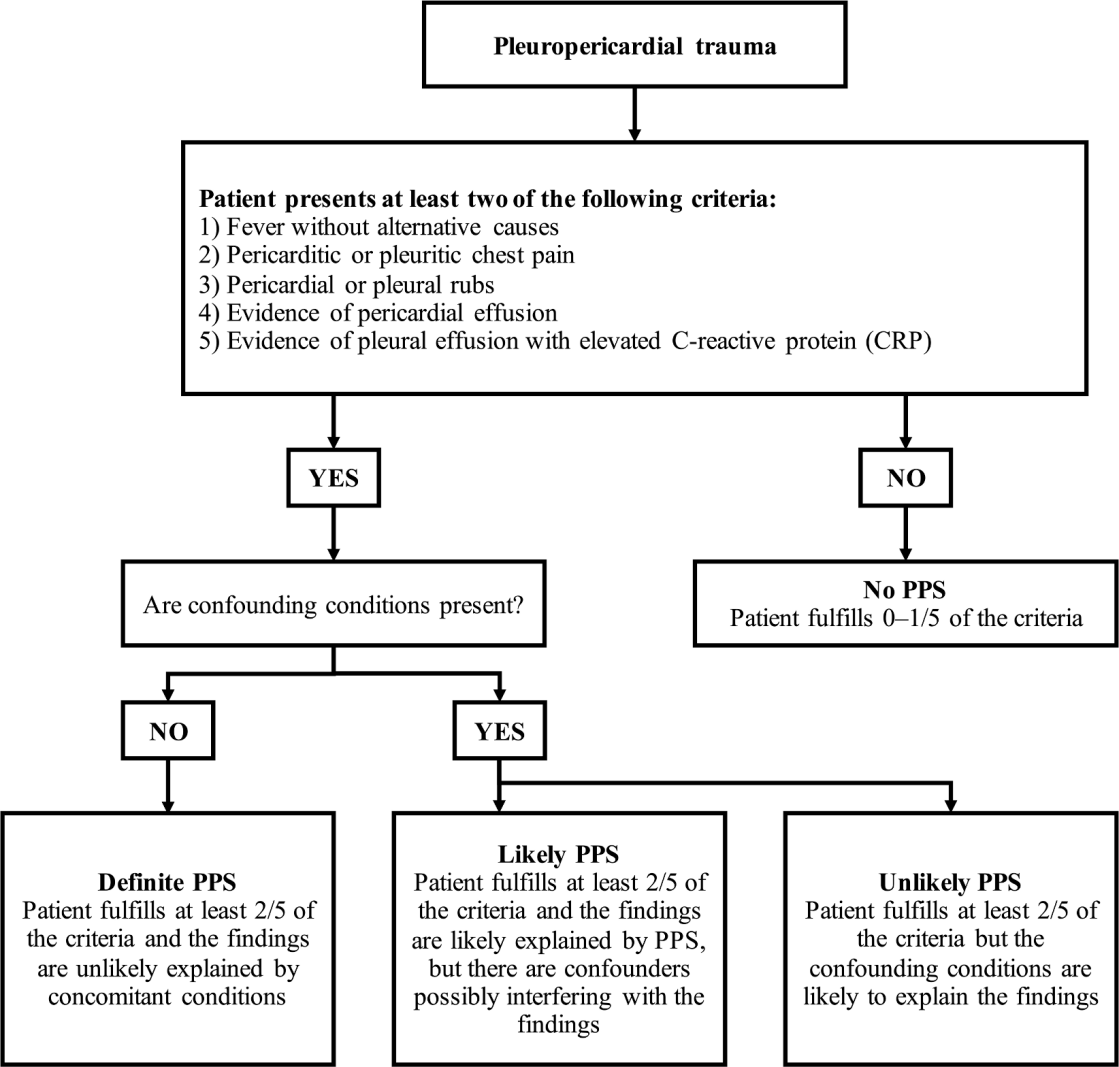
The diagnostic pathway for postpericardiotomy syndrome (*PPS*).

The diagnosis of PPS was determined on the basis of 4 categories: “definite PPS,” “likely PPS,” “unlikely PPS,” and “no PPS.” The diagnosis was made in 2 steps (Figure 1). In the first step, patients presenting at least 2 of the following criteria were included: (1) fever without alternative causes, (2) pericarditic or pleuritic chest pain, (3) pericardial or pleural rubs, (4) evidence of pericardial effusion, and/or (5) evidence of pleural effusion with elevated C-reactive protein (CRP) (ESC PPS criteria^13^). In the second step, the patients fulfilling the criteria were divided into the 4 categories according to the following: (1) definite PPS: the patient fulfills the criteria and the findings are unlikely explained by concomitant conditions; (2) likely PPS: the patient fulfills the criteria and the findings are likely explained by PPS, but there are confounders possibly interfering with the findings; (3) unlikely PPS: the patient fulfills some or all of the criteria but the confounding conditions are likely to explain the findings; and (4) no PPS: the criteria are not fulfilled. The diagnosing process was executed by dedicated PPS researchers using patient records and all other available data. The subcategorization was determined on the basis of an assessment of laboratory and imaging findings, clinical presentation, potential confounding conditions, and therapeutic response. The tests and other examinations for differential diagnostics were executed as clinically indicated. Relapse was defined as worsening pericardial or pleural effusion while on medication or after the withdrawal of medication.

Diabetes, dyslipidemia, and hypertension were defined as a disease requiring medical treatment and chronic lung disease as a pulmonary disease requiring the long-term use of bronchodilators or steroids. Peripheral arterial disease was defined as 1 or more of the following: claudication, carotid artery disease of >50% diameter, and previous or planned intervention on the abdominal aorta, limb arteries or carotids. Heavy alcohol consumption was defined as >14 standard drinks a week for women and >21 standard drinks a week for men. Previous cardiac surgery was defined as 1 or more previous major cardiac operation involving opening of the pericardium. Urgent operation was defined as an operation performed during the index hospital stay, emergency operation as an operation before the next working day, and salvage procedure as an operation in which patients require cardiopulmonary resuscitation en route to the operating theater or before the induction of anesthesia. In-hospital new-onset atrial fibrillation (NOAF) was confirmed by a 12-lead electrocardiographic recording or telemonitoring indicating an AF episode of 5 minutes or longer postoperatively without previous recorded AF or atrial flutter. Ischemic stroke was defined as a permanent focal neurologic deficit adjudicated by a neurologist and confirmed via computed tomography or magnetic resonance imaging. Only ischemic strokes considered definite by the treating neurologist or physician were included in the present study. Pneumonia was defined as symptoms of infection (fever, malaise), elevation of CRP or leukocyte count (excluding leg wound infection), and/or imaging finding consistent with infection (radiograph, computed tomography, magnetic resonance imaging, or ultrasound). Mediastinitis was defined as symptoms of infection (fever, malaise), elevation of CRP or leucocyte count (excluding leg wound infection), evident purulent wound secretion or abscess, and/or imaging finding consistent with infection at the mediastinal area and without signs of alternative diagnoses with similar presentation. Reoperation for bleeding was defined as resternotomy because of bleeding.

The study protocol was approved by the ethical committee of the Hospital District of Southwest Finland and the ethics committee of the National Institute for Health and Welfare (Finland) (ETMK:65/1801/2015 236§, received May 2015). The study adheres to the Declaration of Helsinki. Written informed consent was obtained from the study subjects. The data that support the findings of this study are available from the corresponding author upon reasonable request.

### Statistical Analysis

Statistical analyses were conducted with IBM SPSS Statistics software, version 29.0.1, and R statistics software, version 4.4.3 (R Foundation for Statistical Computing). Continuous variables are reported as mean ± standard deviation if normally distributed and as median (25th-75th percentiles) if skewed. The data were tested for normal distribution using the Shapiro-Wilk test. Categorical variables are described as counts and percentages. To evaluate the association between baseline factors and PPS categories, univariable multinomial logistic regression was performed. The “no PPS” category was used as a reference category. Differences in clinical findings of PPS were assessed using the χ^2^ test and Fisher exact test. The survival estimates were conducted using Kaplan-Meier method, and the associations between PPS classes and mortality were assessed using Cox proportional hazards regression univariable model. The proportional hazards assumption was evaluated using Schoenfeld residuals. Multiple testing correction was not applied due to the exploratory nature of the study.

## Results

### Incidence of PPS

**Figure 2:**
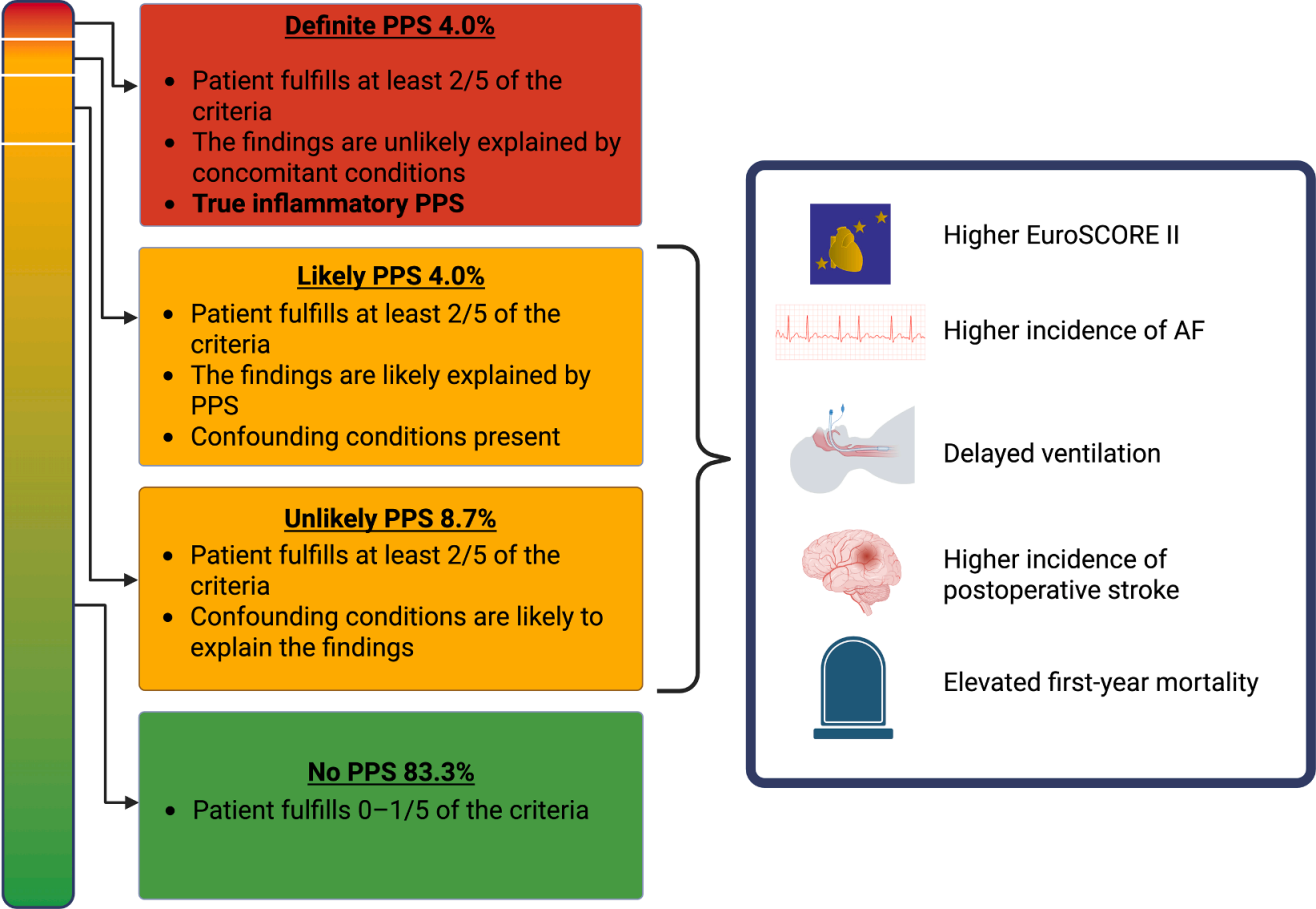
Incidence of different postpericardiotomy syndrome (*PPS*) categories after open-heart cardiac or ascending aorta surgery. *EuroSCORE*, European System for Cardiac Operative Risk Evaluation; *AF*, atrial fibrillation.

A total of 1001 patients were enrolled during the study period, of whom 991 underwent cardiac surgery involving the opening of the pericardium. The median follow-up time after the operation was 5.0 (4.0-6.9) years. The follow-up for PPS was complete for 99.2% of the patients. Overall, the diagnostic criteria of PPS were fulfilled in 164 (16.7%) patients. Upon further classification, “definite PPS” was found in 39 (4.0%) patients and “likely PPS” similarly in 39 (4.0%) patients. Furthermore, 86 (8.7%) patients presented “unlikely PPS” and 819 (83.3%) “no PPS” (Figure 2). The median time to diagnosis was 42 (19-63) days for “definite PPS” and 24 (9-49) days for “likely PPS.” We analyzed the requirement of invasive interventions separately in both groups. “Definite PPS” required pleural drainage in 11 of 39 (28.2%) patients, pericardial drainage in 9 of 39 (23.1%) patients, and led to cardiac tamponade in 4 of 39 (10.3%) patients. In the “likely PPS” group, 12 of 39 (30.8%) patients required pleural drainage and 1 of 39 (2.6%) developed cardiac tamponade, which was managed with pericardial drainage. In addition, a single case required delayed resternotomy attributable to early postoperative pericardial effusion and serous secretion from the drains.

The baseline characteristics and in-hospital events in the PPS categories are detailed in Table 1. Patients with “definite PPS” or “likely PPS” had greater body mass index. In addition, patients with a greater European System for Cardiac Operative Risk Evaluation II percentage were more often categorized into “likely PPS” or “unlikely PPS” categories, and patients in the “unlikely PPS” category had a greater incidence of postoperative stroke. Furthermore, the incidence of “definite PPS” was lower in patients undergoing coronary artery bypass grafting, and “likely PPS” was more common in patients undergoing mitral valve replacement. In the analysis of in-hospital outcomes, patients in the “likely PPS” and “unlikely PPS” categories developed NOAF more often, and delayed ventilation was less common in the “no PPS” category. Furthermore, stroke was more common among patients in the “unlikely PPS” category.

**Table 1:** Baseline characteristics and in-hospital events in different PPS categories

Characteristic	Definite PPS (n = 39)	Likely PPS (n = 39)	Unlikely PPS (n = 86)	No PPS (n = 819)	*P* value
Age, y	66.0 (53.0-72.0)	68.0 (61.0-72.0)	67.5 (62.0-73.0)	68.0 (60.0-77.0)	.455
Female	12 (30.8%)	7 (17.9%)	23 (26.7%)	186 (22.7%)	.473
Diabetes	8 (20.5%)	12 (30.8%)	24 (27.9%)	213 (26.0%)	.744
Type 1	0 (0.0%)	3 (7.7%)	6 (7.1%)	26 (3.2%)	.072
Type 2	8 (20.5%)	9 (23.1%)	17 (20.0%)	183 (22.5%)	.949
Dyslipidemia	20 (51.3%)	30 (76.9%)	53 (61.6%)	556 (67.9%)	.062
Hypertension	25 (64.1%)	26 (66.7%)	65 (75.6%)	567 (69.2%)	.521
Peripheral arterial disease	0 (0.0%)	2 (5.1%)	5 (5.9%)	45 (5.5%)	.224
Coronary artery disease	14 (35.9%)	22 (56.4%)	46 (53.5%)	442 (54.0%)	.167
Paroxysmal atrial fibrillation	3 (7.9%)	8 (20.5%)	15 (17.4%)	97 (11.9%)	.186
Chronic atrial fibrillation	5 (13.2%)	5 (12.8%)	9 (10.5%)	65 (8.0%)	.489
Chronic lung disease	5 (12.8%)	7 (17.9%)	12 (14.0%)	105 (12.8%)	.838
Active smoking	5 (13.2%)	2 (5.3%)	9 (10.5%)	115 (14.1%)	.292
Active or ex-smoker	16 (42.1%)	14 (35.9%)	38 (44.2%)	370 (45.3%)	.689
Body mass index, kg/m^2^	28.5 (24.9-31.6)	28.7 (24.9-34.8)	27.0 (24.0-30.5)	27.6 (24.7-30.8)	.042
Heavy alcohol consumption	2 (5.3%)	1 (2.7%)	2 (2.3%)	31 (3.9%)	.813
Previous stroke	5 (12.8%)	3 (7.7%)	13 (15.1%)	59 (7.2%)	.088
Previous myocardial infarction	5 (12.8%)	7 (17.9%)	17 (19.8%)	114 (13.9%)	.491
Previous percutaneous coronary intervention	5 (12.8%)	5 (12.8%)	9 (10.5%)	110 (13.4%)	.887
Previous cardiac surgery	0 (0.0%)	1 (2.6%)	2 (2.3%)	34 (4.2%)	.255
Active endocarditis	0 (0.0%)	0 (0.0%)	0 (0.0%)	6 (0.7%)	.532
Previous endocarditis	1 (2.6%)	0 (0.0%)	2 (2.3%)	4 (0.5%)	.229
Previous venous thromboembolism	0 (0.0%)	0 (0.0%)	0 (0.0%)	0 (0.0%)	.257
EuroSCORE II, %	1.6 (0.97-2.9)	2.0 (1.2-3.0)	2.1 (1.1-3.2)	1.5 (1.0-2.4)	.011
Pulmonary artery hypertension					
Moderate to severe (systolic ≥31 mm Hg)	14 (37.8%)	11 (30.6%)	33 (41.3%)	279 (38.5%)	.741
Severe (systolic >55 mm Hg)	1 (2.7%)	1 (2.8%)	1 (1.3%)	23 (3.2%)	.762
NYHA class III or greater	8 (20.5%)	7 (17.9%)	23 (26.7%)	134 (16.4%)	.137
Preoperative eGFR, mL/min/1.73 m^2^	77.6 (58.7-95.7)	74.8 (59.1-93.6)	74.2 (62.6-91.4)	78.1 (64.1-90.9)	.791
Urgent, emergency, or salvage procedure	6 (15.4%)	7 (17.9%)	17 (19.8%)	153 (18.7%)	.946
Procedure type					
CABG	13 (33.3%)	24 (61.5%)	46 (53.5%)	472 (57.6%)	.022
AVR	18 (46.2%)	17 (43.6%)	28 (32.6%)	241 (29.5%)	.054
MPL	7 (17.9%)	4 (10.3%)	12 (14.0%)	89 (10.9%)	.527
MVR	1 (2.6%)	7 (17.9%)	4 (4.7%)	46 (5.6%)	.043
Ascending aortic surgery	9 (23.1%)	4 (10.3%)	11 (12.8%)	80 (9.8%)	.112
Other than isolated CABG	26 (66.7%)	15 (38.5%)	39 (45.9%)	339 (41.8%)	.019
In-hospital outcomes					
New-onset atrial fibrillation	11 (36.7%)	14 (53.8%)	36 (57.1%)	243 (36.8%)	.007
Cardioversion	0 (0.0%)	2 (5.1%)	4 (4.7%)	20 (2.5%)	.257
Deep wound infection	0 (0.0%)	1 (2.6%)	1 (1.2%)	3 (0.4%)	.402
Mediastinitis	0 (0.0%)	0 (0.0%)	1 (1.2%)	4 (0.5%)	.721
Pneumonia	0 (0.0%)	1 (2.6%)	5 (5.8%)	17 (2.1%)	.149
Stroke	1 (2.6%)	1 (2.6%)	6 (7.0%)	12 (1.5%)	.045
Delayed ventilation	6 (15.4%)	6 (15.4%)	12 (14.1%)	52 (6.4%)	.011
Acute de novo dialysis	1 (2.6%)	1 (2.6%)	0 (0.0%)	6 (0.7%)	.340
Reoperation due to bleeding	2 (5.1%)	3 (7.7%)	7 (8.1%)	45 (5.5%)	.763
Reoperation due to infection	0 (0.0%)	0 (0.0%)	1 (1.2%)	5 (0.6%)	.730
Reoperation due to valve problem	0 (0.0%)	0 (0.0%)	1 (1.2%)	0 (0.0%)	.181
Reoperation, any	2 (5.1%)	3 (7.7%)	9 (10.5%)	48 (5.9%)	.462
Length of hospital stay, d	8.0 (7.0-10.0)	8.0 (7.0-10.3)	9.0 (7.0-14.0)	8.0 (7.0-10.0)	<.001

Continuous variables are reported as median (interquartile range) or mean ± standard deviation. Values in parentheses are percentages. *PPS*, Postpericardiotomy syndrome; *EuroSCORE*, European System for Cardiac Operative Risk Evaluation; *NYHA*, New York Heart Association; *eGFR*, estimated glomerular filtration rate; *CABG*, coronary artery bypass graft; *AVR*, aortic valve replacement; *MPL*, mitral valve plasty; *MVR*, mitral valve replacement.

### Clinical Findings of PPS

The clinical findings in “definite PPS” and “likely PPS” categories are detailed in Table 2. No statistically significant differences were found between the 2 groups. No pericardial constriction was found in either of the groups during follow-up.

**Table 2:** Clinical findings of PPS

Characteristic	Definite PPS (n = 39)	Likely PPS (n = 39)	*P* value
Elevated CRP (>10 mg/L)	31 (79.5%)	28 (75.7%)	.690
Echocardiogram: pericardial effusion	37 (94.9%)	30 (81.1%)	.063
Chest radiograph: pleural effusion	25 (65.8%)	27 (75.0%)	.386
Chest radiograph: cardiac silhouette enlargement	16 (42.1%)	11 (30.6%)	.302
ECG: widespread ST-elevations	4 (11.8%)	8 (26.7%)	.127
Tamponade	4 (10.3%)	1 (2.6%)	.358
PPS relapse	4 (10.3%)	2 (5.1%)	.675

*PPS*, Postpericardiotomy syndrome; *CRP*, C-reactive protein; *Echo*, echocardiogram; *ECG*, electrocardiogram.

### Treatment of PPS and Relapses

First-line medications for “definite PPS” included nonsteroidal anti-inflammatory drugs in 17.9%, colchicine in 59.0%, and corticosteroids in 20.5% of the patients. Similarly, first-line medications for “likely PPS” included nonsteroidal anti-inflammatory drugs in 0%, colchicine in 7.7%, and corticosteroids in 10.5% of the patients. There were 4 (10.3%) relapses in the “definite PPS” group and 2 (5.1%) in the “likely PPS” group.

### PPS and Mortality

**Figure 3:**
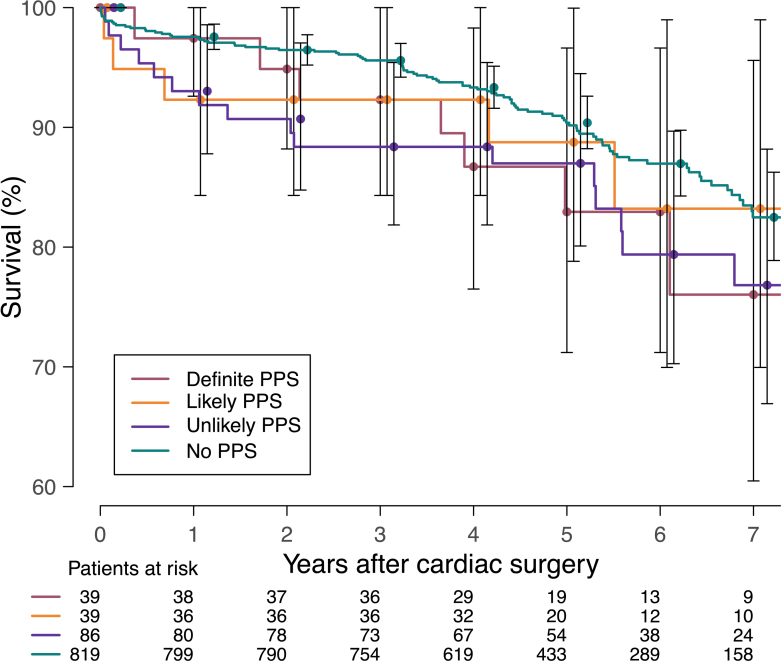
Survival in different postpericardiotomy syndrome (*PPS*) categories with 95% CIs after open-heart surgery.

Median follow-up time for mortality was 5.6 (4.2-7.1) years and overall 129 deaths occurred during the follow-up. Mortality in different PPS categories were the following: “definite PPS” 7 of 39 (17.9%), “likely PPS” 5 of 39 (12.8%), “unlikely PPS” 16 of 86 (18.6%), and “no PPS” 94 of 819 (11.5%). Survival after open-heart surgery in different PPS categories is shown in Figure 3. Although no statistically significant differences in long-term mortality were found, patients classified to “likely PPS” or “unlikely PPS” groups had greater mortality at 1 year compared with “no PPS” group (hazard ratio, 3.00; 95% CI, 1.36-6.58, *P* = .006).

## Discussion

The present study demonstrates that the existing diagnostic criteria for PPS defined in the 2025 ESC Guidelines^13^ are frequently met by confounding conditions that are unlikely to be related to the syndrome's presumed inflammatory etiology. Notably, only approximately one half of patients meeting the ESC criteria were classified as having either “definite PPS” or “likely PPS,” underscoring the limitations of current diagnostic standards in routine clinical practice.

The incidence of “definite PPS” in this study was 4.0%, markedly lower than the 9 to 29% reported in recent studies.^5^, ^6^, ^7^, ^8^, ^9^^,^^11^^,^^12^ This discrepancy likely reflects the inclusion of patients with overlapping or confounding conditions in previous research, as those studies applied the ESC criteria without stratification. This suggests potential limitations in applying the findings of previous studies to everyday clinical practice. The current diagnostic criteria for PPS were first introduced in the ESC guidelines in 2015.^14^ The diagnostic criteria are derived from clinical trials conducted by Imazio and colleagues^7^^,^^15^ and by Finkelstein and colleagues.^16^ When combining the “definite,” “likely,” and “unlikely” PPS categories, the overall incidence of patients fulfilling the current diagnostic criteria was 16.7% in this study, aligning with previously reported rates and highlighting the need for more refined diagnostic tools.^7^^,^^12^

Confounding conditions, such as congestive heart failure, systemic inflammatory response, and postoperative complications, frequently obscure the diagnosis of PPS, particularly in patients with multiple comorbidities. These patients, categorized as “likely PPS” or “unlikely PPS,” exhibited greater European System for Cardiac Operative Risk Evaluation II values, increased incidence of NOAF, delayed postoperative ventilation, greater incidence of postoperative stroke, and elevated first-year mortality. The findings suggest that the current ESC criteria may lack specificity, leading to potential overdiagnosis and overtreatment of PPS.

Surgical complexity also influenced PPS categorization. Patients undergoing mitral valve replacement and aortic procedures—typically more extensive than coronary artery bypass grafting—had a greater incidence of “likely PPS,” consistent with previous studies linking increased surgical trauma to PPS development.^4^^,^^11^^,^^17^^,^^18^ These findings reinforce the importance of considering procedural factors and patient complexity when diagnosing PPS. The elevated body mass index in patients with “definite PPS” and “likely PPS” may be explained by the proinflammatory state commonly associated with obesity.^19^

The 2-step diagnostic pathway introduced in the present study reflects real-world clinical practice more accurately. Furthermore, this diagnostic process enables comparative analysis between “definite PPS” and “no PPS” cases, thereby supporting further investigations into the inflammatory etiology of PPS and eventually targeted therapeutic approaches.

### Clinical Implications

Although the recommended diagnostic criteria were first introduced in the ESC guidelines in 2015,^14^ these have shown limited value in clinical settings, primarily as the result of concerns regarding specificity. The study revealed that “definite PPS” accounts for only one quarter of the cases that clinically meet the recommended PPS criteria, and even when combined with “likely PPS,” they account for only half of such cases. This indicates that most of the PPS diagnoses reported in previous prospective studies are clinically irrelevant, which also aligns with the findings of a previous study.^10^ The nonspecificity and broad inclusivity of the currently recommended PPS criteria may result in overdiagnosing and overtreatment of PPS, although patients in the “likely PPS” and, as expected, also in the “unlikely PPS” categories often recover without anti-inflammatory medication. Moreover, the diagnostic pathway applied in this study separates the “definite PPS” and “no PPS,” which allows the comparison between these 2 groups in future studies, making it possible to investigate more specifically the PPS cases driven by the underlying inflammatory mechanism. This is essential for advancing knowledge of the pathophysiology of the disease, and, ultimately for enabling the development of specific pharmacologic treatments and preventive strategies. Most patients in the “likely PPS” group are likely to have true inflammatory PPS, whereas its prevalence in the “unlikely PPS” group is expected to be markedly lower. Confirmation, however, requires further investigation of the “definite PPS” group, for example by identifying a suitable biomarker for diagnostic use.

### Strengths and Limitations

CAREBANK is a prospective study focused on the adverse events after cardiac surgery. In addition to the prospective nature of the database, another strength of this database is that a validated, structured case report form was used in data collection. The follow-up for PPS was complete for as much as 99.2% of the patients, because the treatment of patients belonging to the catchment areas of the participating institutions is mainly centralized. This enables the collection of detailed information on major clinical events requiring hospitalization, as well as information on postoperative outpatient visits, because these services were primarily performed in tertiary health care. The diagnosing process was executed by dedicated PPS researchers, which confirms the reliability of the diagnoses. Data on late mortality were obtained from the Statistics Finland, which ensures the quality of survival data of the patients even if the patient moved. The diagnostic tests for PPS, such as chest x-ray and echocardiography, were not performed on a routine basis to all patients (eg, on a weekly basis), which might cause underdiagnosing of PPS. Nevertheless, the tests were performed as clinically indicated, reflecting the “real-world” feature of the present study. Although the diagnostic approach presented in this study enhances the identification of PPS and provides a more accurate representation of real-world clinical practice, some uncertainty remains regarding the reliability of the “definite PPS” and “no PPS” classifications, as no definitive diagnostic test for the condition is currently available.

## Conclusions

This study suggests that the majority of PPS diagnoses reported in previous prospective studies were likely driven by confounding factors, whereas the classic delayed inflammatory form accounts for only a fraction of cases. We propose a 4-tier subclassification of PPS on the basis of the clinical relevance of findings, reflecting real-world practice. Adopting this framework in future research could enhance the clinical applicability of emerging evidence.

## Conflict of Interest Statement

T.K. reports grants from EU and Business Finland (Moore4Medical), EU/EIC Pathfinder, Finnish Medical Foundation, Finnish Foundation for Cardiovascular Research, and State Research Funding, Vifor Pharma, and AtriCure. J.G. reports a grant from Vifor Pharma. All other authors reported no conflicts of interest.

The *Journal* policy requires editors and reviewers to disclose conflicts of interest and to decline handling or reviewing manuscripts for which they may have a conflict of interest. The editors and reviewers of this article have no conflicts of interest.
